# Predominance of girls with cancer in families with multiple childhood cancer cases

**DOI:** 10.1186/s12885-017-3899-8

**Published:** 2017-12-19

**Authors:** Karl-Johan Stjernfelt, Kristoffer von Stedingk, Thomas Wiebe, Lars Hjorth, Håkan Olsson, Ingrid Øra

**Affiliations:** 1 Department of Pediatrics, Pediatric Oncology and Hematology, Lund University, Skane University Hospital, Lund, Sweden; 2 Translational Cancer Research, Medicon Village, Lund University, Lund, Sweden; 3 Department of Oncology, Lund University, Skane University Hospital, Lund, Sweden; 4 Department of Pediatrics, Clinical Sciences, Lund University, Skane University Hospital, 22185 Lund, Sweden

**Keywords:** Pediatric cancer, Familial cancer predisposition, Hereditary cancer syndrome, Genetic cancer susceptibility

## Abstract

**Background:**

Recent studies indicate that one of four childhood cancers can be attributed to hereditary genetic abnormalities.

**Methods:**

The Lund Childhood Cancer Genetic study includes newly diagnosed childhood cancer patients as well as childhood cancer survivors visiting the Department of Pediatrics or the Late Effect Clinic at Skåne University Hospital, Lund, Sweden. Questionnaires regarding family history of cancer and blood samples were provided. Reported data were validated and extended by use of the Swedish Population- and Cancer Registries. Demographics in families with one case of childhood cancer (FAM1) were investigated and compared to families with multiple cases of childhood cancer (FAM > 1) as well as to childhood cancer in the general population.

**Results:**

Forty-one out of 528 families (7.8%) had more than one case of childhood cancer. In 23 families the affected children were relatives up to a 3rd degree (4.4%). In FAM > 1, 69.2% of the children with leukemia and 60% of those with tumors in the central nervous system (CNS) had a childhood relative with matching diagnosis, both significantly higher than expected. Significantly more female than male patients were observed in FAM > 1 compared to FAM1. This female predominance was most striking in childhood leukemia (77% female) and also, yet to a lesser extent, in CNS tumors (68% female).

**Conclusions:**

We conclude that the high proportion of children with leukemia or CNS tumors in FAM > 1 having a childhood relative with the same diagnosis suggests a hereditary background. Moreover, we report a female predominance in childhood leukemia and childhood CNS tumors in FAM > 1, which may indicate a hereditary gender-specific risk factor in these families.

## Background

The increased cancer risk amongst relatives of childhood cancer patients has been reported in several studies over the past decades. Although the reports are varying, they generally show an increased risk for cancer for both siblings [1–3] and parents [2–8]. In 1991, genetic conditions could explain 3.07% of childhood cancer cases but that number increased to 4.2% when data from family history was included [9]. More recent studies estimate heredity to account for 29% of childhood cancer cases [10, 11]. Several studies confirm that earlier onset cancers have a hereditary component [3, 4, 12–14], which is more pronounced if the child has a central nervous system (CNS) tumor [4–7]. In addition, women in families with childhood cancer have an increased cancer incidence, especially with regard to breast cancer [2, 4–7, 12]. A recent genome sequencing study of pediatric cancer cases showed that 8.5% had germline mutations in known cancer predisposition genes yet no obvious association to familial history of cancer up to second degree was seen [15]. Furthermore, many of the studies mentioned above showed that even when excluding already known familial syndromes an increased risk of cancer remained [1, 2, 4, 5, 12].

Multiple primary cancers are common in hereditary syndromes such as familial Wilms tumor and heritable retinoblastoma, where multifocal and bilateral tumors in paired organs are often observed [16–18]. Down syndrome is associated with an increased risk of acute leukemia during childhood with a ten- to twenty-fold higher risk than in the general population [19, 20]. The number of rare pediatric cancer syndromes is increasing and the importance of early cancer surveillance strategies to reduce morbidity and mortality is extensively debated in the pediatric oncology community [21, 22]. Clinically evident hereditary syndromes have, to a large extent, already been defined and genetically explained, however, subclinical syndromes and/or hereditary genetic aberrations may yet to be discovered. Taking this into consideration, studies using high-throughput genetic techniques characterizing subclinical genetic predisposition to childhood cancers are of great importance.

In the current study, we present work from the Lund Childhood Cancer Genetic (LCCG) study, aimed at investigating possible genetic predispositions in families with reported cases of childhood cancer. In the LCCG-study, data on family history of cancer and blood samples are collected from childhood cancer patients and childhood cancer survivors in the southern healthcare region of Sweden. The overall aim of this on-going study is to characterize cancer predisposing aberrations and/or associations through in depth germ-line analyses with correlation to detailed and verified family history of cancer. With focus on families with multiple cases of childhood cancer (FAM > 1), we describe characteristics in terms of age at diagnosis, sex, diagnoses and diagnosis distribution. Here we identify families with potential previously undiscovered hereditary syndromes, laying ground for future genetic analyses, which could shed light on novel hereditary factors.

## Methods

The Lund Childhood Cancer Genetic (LCCG) study was initiated in 2008. Children with malignancies diagnosed, treated and followed at the Pediatric Oncology and Hematology ward, as well as childhood cancer survivors visiting the Late Effect Clinic at the Department of Oncology, Skåne University Hospital in Lund, Sweden are offered to participate.

The eligibility criteria for patient inclusion from the Pediatric Department are 1) diagnosis before 19 years of age with a malignancy with codes 140–209 according to the International classification of diseases 7th edition (ICD-7) *and* from the Late Effect Clinic 2) diagnosis since 1 January 1970. Blood samples from both the patients and parents are collected. Patients and parents are requested to complete a standardized self-reported questionnaire, querying for name, date of birth and the national identification number, history of cancer amongst first, second, and third degree relatives. Information regarding specific type of cancer and date of/age at diagnosis inclusive outcome (if fatal, date of death) for each relative with a history of cancer is obtained. In addition, questions about cancer in more distant relatives are included. The questionnaire is explained and handed out by the study nurse/physician and returned by mail. Although all types of cancers (including adult) were reported, only childhood cancers were included and examined in the current study.

Pedigrees were created for every family with the patient included in the LCCG study as *study patient*, using the program Progeny 9 (Progeny Software, LLC).

The Population Registry in Sweden was used to 1) collect and/or validate the identification of all patient’s relatives living in Sweden and to 2) extend pedigrees to include all relatives of the chosen degree and thereby supplement data lacking from questionnaires. All participants were crosschecked with the Population Registry for data on vital status, and the Swedish Cancer Registry to confirm reported cancer diagnosis or to identify any potential relatives with unreported cancer diagnoses. For relatives living abroad, only questionnaire-based information was available.

Pathology reports and patient charts were reviewed for study patients and any relative with a childhood cancer in order to validate the diagnosis and to check for possible hereditary syndromes and other cancer predisposing factors. Eight childhood cancer cases in relatives could not be verified microscopically due to diagnosis either before 1970 or outside of Sweden. Three patients were diagnosed with unspecified leukemia. Due to unspecified diagnoses and the relatively small cohort, all leukemia diagnoses inclusive ALL and acute myeloid leukemia (AML) were grouped together for subsequent analyses. We further investigated the cytogenetic subtype and risk group of the acute lymphatic leukemia (ALL) cases in the study.

Following data collection of all childhood cancer cases, FAM > 1 were identified for characterization in terms of age at diagnosis, sex, diagnosis and diagnosis distribution. All relatives to the third degree were included. When childhood cancer cases in relatives of a higher degree were reported, the pedigree was expanded to ensure correct degree of relationship with the study patient.

The NORDCAN database of all childhood cancer patients from all Nordic countries (1970–2013) [23] was used for demographic comparisons with childhood cancer in the general population. This database lacked information regarding age at diagnosis, therefore we used the data of age at diagnosis from a recent epidemiological review of European childhood cancer databases for comparative statistics [24].

### Statistical analysis

**Fig. 1:**
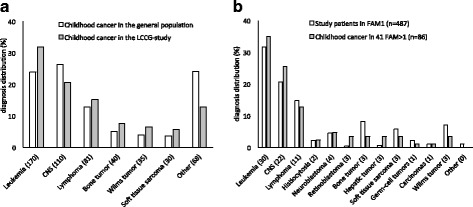
Diagnosis distribution of childhood cancer cases in **a** Lund Childhood Cancer Genetic (LCCG) study (grey)(*n* = 534) compared to the childhood cancer population in the Nordic countries [23] (white). The value in brackets represents the number of study patients in the LCCG-study with corresponding diagnosis. **b** Families with multiple childhood cancers (FAM > 1, *n* = 86 in 41 families) (grey) compared to families with one childhood cancer case (FAM1, *n* = 487) (white) in the LCCG-study. The value in brackets represents the number of study patients and relatives with corresponding diagnosis in FAM > 1. The higher number of leukemia cases and lower number CNS cases in the LCCG cohort compared to the general population were not significant after correction for multiple testing

The statistical software SPSS 22.0 was used for statistical analyses. Pearson’s chi-squared test was used to compare categorical data (gender and diagnosis) between childhood cancer affected individuals in families with one case of childhood cancer (FAM1) and FAM > 1. For categorical data with smaller samples (*n* < 5) Fisher’s exact test was used. Student T test was used to compare mean age at diagnosis. All analyses were two-sided and *p*-values <0.05 were considered significant. False discovery rate (FDR) correction was applied to account for multiple testing between patient groups (comparisons limited to patient groups with *n* > 5) regarding diagnostic distribution and gender (Fig. 1 and Table 3).

## Results

As of October 2014, 534/679 (78.6%) patients in the study had returned the questionnaires with 31 (4.6%) patients actively declining participation. Approximately half of the study patients were included from the Pediatrics Department and half from Late Effect Clinic.

Forty-seven study patients in the LCCG-study, 8.8% of 534 (95% CI: 6.4–11.2%), had a relative with childhood cancer. Six study patients were related to another study patient that was already included in the study. These were classified as relatives in subsequent analyses. Accordingly, 41 study patients, 7.8% of 528 (95% CI: 5.5–10.0%) had a relative diagnosed with childhood cancer. In four families there were three cases of childhood cancer. The 41 families with more than one childhood cancer (FAM > 1) with a total of 86 children are described in Table 1. Two of these families had relatives of the 6th degree with a childhood cancer diagnosis, while the remaining 39 families had relatives up to the 5th degree (Table 1). In 23 of these families (4.4%, 95% CI: 2.6–6.1%) the study patient had a 1st to 3rd degree relative with a childhood cancer diagnosis.

**Table 1:** Characteristics of study patients and corresponding relatives in families with more than one childhood cancer (FAM > 1)

Study patient			Relative of study patient				
Nr	Diagnosis	Gender	Age (years)	Diagnosis	Gender	Age (years)	Degree of relative
*Leukemia*							
1	ALL	m	4.3	ALL	m	4.2	5
				ALL	f	3.9	5
2	ALL	f	2.1	ALL	f	5.6	5
				ALL	f	7.5	5
3	ALL	f	6.0	ALL	f	14.1	3
4	ALL	f	4.4	ALL	f	5.3	1
5	ALL	f	2.3	ALL	f	1.3	4
6	ALL	f	3.2	Leukemia^a^	f	13.0	2
7	ALL	f	8.1	AML	m	17.7	2
8	ALL	f	3.4	Hodgkin lymphoma	m	10.5	4
9	ALL	f	2.1	Ganglioglioma	m	7.0	1
10	ALL	f	8.6	Ewing sarcoma	f	8.2	5
11	ALL	f	1.6	Retinoblastoma	f	2.1	3
12	AML	f	12.4	ALL	f	4.5	4
13	AML	f	2.3	AML	m	0.3	4
*CNS tumors*							
14	Ependymoma	m	11.7	High grade glioma	f	5.1	3
15	Ependymoma	m	6.8	High grade glioma	f	5.1	4
16	Ependymoma	f	10.2	CNS tumor^a^	f	13.1	2
17	Astrocytoma	m	1.6	Neuroblastoma	f	0.9	1
18	Astrocytoma	f	16.0	CNS undefined	f	7.1	5
19	Astrocytoma	f	14.0	Hodgkins lymphoma	f	14.2	3
20	Optic tract glioma	f	4.6	Ependymoma	f	0.8	4
21	Optic tract glioma	m	2.2	Wilms tumor	f	1.6	6
22	Ganglioglioma	f	14.9	Astrocytoma	m	14.5	2
23	Adenoma hypophysis	f	12.8	Neuroblastoma	m	2.6	3
*Lymphomas*							
24	Hodgkin lymphoma	m	8.3	ALL	f	5.6	4
25	Hodgkin lymphoma	m	13.5	ALL	f		4
26	Hodgkin lymphoma	f	15.3	Wilms Tumor	f	13.2	3
				Ewing sarcoma	m	13.2	3
27	Hodgkin lymphoma	f	17.0	CNS tumor^a^	m	0.0	3
28	Burkitt lymphoma	m	14.5	Astrocytoma	f	0.9	5
29	Burkitt lymphoma	m	16.1	Rhabdomyosarcoma	m	9.1	2
30	Anaplastic large cell lymphoma	m	12.9	Hepatoblastoma	m	0.9	5
31	Diffuse large B-cell lymphoma	m	16.5	AML	m	8.0	3
*Other diagnoses*							
32	Hepatoblastoma	f	2.6	Hodgkin lymphoma	m	11.7	3
33	Langerhans cell histiocytosis	m	4.3	Langerhans cell histiocytosis	m	2.7	1
34	Wilms tumor	m	3.5	ALL	m	5.3	2
35	Neuroblastoma	f	0.3	CNS tumor^a^	f	9.8	6
36	Ganglioneuroblastoma	f	2.7	Osteosarcoma	f	19.0	2
37	Rhabdomyosarcoma	m	2.2	CNS tumor^a^	f	6.0	5
				Malign melanoma^a^	f	17.5	4
38	Rhabdomyosarcoma	m	4.4	Astrocytoma	f	3.9	5
39	Retinoblastoma	f	0.4	Retinoblastom	m	0.3	1
40	Hepatoblastoma	f	2.5	Leukemia^a^	m	<19^b^	4
41	Dysgerminoma	f	12.6	Leukemia^a^	f	5.0	4

*Abbreviations: ALL* acute lymphatic leukemia, *AML* acute myeloid leukemia, *CNS* central nervous system, *m* male, *f* female, *age* age at diagnosis

^a^microscopic diagnosis not confirmed

^b^exact age unknown

When comparing the diagnosis distribution in the LCCG-study with the diagnosis distribution of childhood cancer in the Nordic countries, we observed a higher percentage of leukemia in the LCCG-study, 32% versus 24% (Fig. 1a). Furthermore, the percentage of CNS tumors was lower than that of the general childhood cancer population, 21% versus 26%, respectively. However, neither of these observations were significant after correction for multiple testing. There was no significant difference between the diagnosis distribution of FAM > 1 and the diagnosis distribution in FAM1 (Fig. 1b).

### High proportion of multiple leukemia and CNS tumors in families with multiple childhood cancers

The 41 FAM > 1 were grouped according to the type of diagnosis of the study patient (Table 1). Out of the 13 study patients with leukemia, nine cases had a relative with childhood leukemia (69.2%) (Table 1), which is significantly higher portion than that of childhood cancer in the general population (*p =* 0*.*001). Two of these families had three cases of childhood ALL. Among patients with CNS tumors, six of the ten study patients (60%) had a relative with a childhood CNS tumor, which is also a higher portion than that of the general population (*p =* 0*.*025). Two families (Family 13 and 14) shared the same relative with a high-grade glioma, however, the two study patients were not related. In subgroup 3 (lymphomas) we found no relative with a childhood lymphoma. Of the four children with neuroblastoma (study patient or relative), three had a relative with a childhood CNS tumor. Four families had cases of both childhood lymphoma and childhood leukemia, of which three cases were Hodgkin’s lymphomas. As for other cancer diagnoses, there were no observed diagnosis patterns (Table 1).

### Age distribution in families with multiple cases of childhood cancer

There was no significant difference in mean age at diagnosis between a) study patients combined with their affected relatives in FAM > 1 (*n* = 86), b) study patients in FAM1 and c) study patients in the LCCG-study (Table 2). In addition, no differences were observed when comparing age at diagnosis between all LCCG-patients with the general childhood cancer population.

**Table 2:** Age at diagnosis of pediatric cancer in families with one - or multiple children with cancer

	Mean age at diagnosis					
	FAM > 1	FAM1		LCCG-study		Gen. Pop.
Diagnosis	years ± SD (n)	years ± SD (n)	*p*	years ± SD (n)	*p*	years [24]
All diagnoses	7.1 ± 5.2 (86)	7.2 ± 5.0 (487)	0.842	7.2 ± 5.1 (534)	0.997	
Leukemia	5.9 ± 4.2 (30)	5.8 ± 4.3 (154)	0.974	5.8 ± 4.2 (170)	0.941	5.0
CNS tumor	7.6 ± 5.1 (22)	8.4 ± 4.5 (100)	0.506	8.4 ± 4.6 (110)	0.449	7.0
Lymphoma	13.7 ± 2.7 (11)	11.0 ± 4.7 (72)	0.067	11.3 ± 4.6 (81)	0.101	10.8
Histiocytosis	3.5 + 1.2 (2)	4.3 ± 3.7 (11)	0.783	4.2 ± 3.5 (12)	0.770	–
Neuroblastoma	1.6 ± 1.2 (4)	2.1 ± 2.7 (22)	0.721	2.1 ± 2.7 (24)	0.740	1.3
Retinoblastoma	0.9 ± 1.0 (3)	1.4 ± 1.0 (2)	0.652	0.9 ± 0.8 (4)	0.935	1.3
Bone tumor	13.4 ± 5.4 (3)	10.8 ± 4.9 (40)	0.370	10.8 ± 4.9 (40)	0.370	11.6
Hepatic tumor	2.0 ± 1.0 (3)	0.7 ± 0.9 (3)	0.179	1.4 ± 1.1 (6)	0.429	1.1
Soft tissue sarcoma	5.3 ± 3.5 (3)	5.7 ± 4.2 (28)	0.863	5.5 ± 4.1 (30)	0.910	6.5
Germ-cell tumors	12.6 (1)	8.9 ± 6.8 (11)	0.611	9.2 ± 6.5 (12)	0.627	8.7
Carcinomas	6.0 (1)	9.2 ± 4.3 (5)	0.532	9.2 ± 4.3 (5)	0.532	111
Wilms’ tumor	6.1 ± 6.2 (3)	3.6 ± 3.3 (34)	0.238	3.6 ± 3.2 (35)	0.231	3.3
Others	- (0)	- (5)	–	- (5)	–	–

*Abbreviations: FAM > 1* families with multiple cases of childhood cancer, *FAM1* families with one case of childhood cancer, *LCCG* Lund Childhood Cancer Genetic, *Gen.Pop* General childhood cancer population, *CNS* central nervous system

### Female predominance in families with multiple childhood cancers

Significantly more female than male childhood cancer patients were observed in FAM > 1, female = 53 (61.6%) and male = 33 (38.4%), than in FAM1, female = 197 (40.5%) and male = 290 (59.5%)(*p =* 0*.*001)(Table 1). The greatest gender difference was found among childhood leukemia cases (relatives with childhood leukemia included) in FAM > 1, female = 23 (76.7%) and male = 7 (23.3%)(*p <* 0*.*001, *FDR p* = 0.004) (Tables 1 and 3).

**Table 3:** Gender distribution of pediatric cancer in families with one - or more children with cancer

Diagnosis	FAM > 1 (n)	FAM1 (n)	LCCG-study (n)	General population [23]
All diagnoses	**0.62** ^a,b^ (86)	1.47 (487)	1.36 (534)	1.2
Leukemia	**0.30** ^a,b^ (30)	1.30 (154)	1.07 (170)	1.2
CNS tumors	**0.47** ^a^ (22)	1.63 (100)	1.50 (110)	1.2
Lymphoma	2.67 (11)	1.67 (72)	1.79 (81)	1.6
Histiocytosis	males only (2)	1.75 (11)	2.00 (12)	–
Neuroblastoma	0.33 (4)	1.75 (22)	1.40 (24)	1.2
Retinoblastoma	0.33 (3)	1.00 (2)	1.00 (4)	1.1
Bone tumor	0.50 (3)	1.50 (40)	1.50 (40)	1.4
Hepatic tumor	0.50 (3)	2.00 (3)	1.00 (6)	1.4
Soft tissue sarcoma	males only (3)	2.50 (28)	2.75 (30)	1.2
Germ-cell tumors	females only (1)	0.83 (11)	0.71 (12)	–
Carcinomas	males only (1)	1.50 (5)	1.50 (5)	–
Wilms tumor	0.50 (3)	1.27 (34)	1.33 (35)	0.9
Others	- (0)	0.25 (5)	0.25 (5)	–

All units are representative of the male-to-female ratio. Significant observations are marked as bold

*Abbreviations: FAM > 1* families with multiple cases of childhood cancer, *FAM1* families with one case of childhood cancer, *LCCG* Lund Childhood Cancer Genetic, *CNS* central nervous system, *FDR* false discovery rate

^a^ *FDR adjusted p <* 0.05 when compared to childhood cancer in FAM1

^**b**^ *FDR adjusted p <* 0.05 when compared to childhood cancer in the general population

Among all 82 female childhood leukemia cases in the LCCG-study, 15 had an additional case of childhood cancer (18.3%, 95% CI: 9.9–26.6%). This observation is significantly higher than the 7.1% risk observed in the rest of the LCCG-study (*p =* 0*.*004) (Table 1). In contrast, of the 88 males with leukemia in the study, only two had a relative with a childhood cancer.

A female predominance was also found among patients with childhood CNS tumors in FAM > 1 compared to FAM1; female = 15 (65.2%) versus male = 8 (34.8) (Tables 1 and 3; *p =* 0*.*010, *FDR p =* 0.020). Among the 44 families with a female case of CNS tumors in the LCCG-study, 13.6% (*n* = 6/44) had two cases of childhood cancer. In families where a male child had a CNS tumor, 7.6% (5/66) had an additional case of childhood cancer.

The male-to-female ratio in the LCCG-study was in line with that of the childhood cancer in the general population except for soft tissue sarcoma where the LCCG-study had a higher ratio (*p =* 0.033, *FDR p* = 0.297) (Table 3). This observation, however, was not significant after adjusting for multiple testing. Interestingly, the male-to-female ratio of all identified individuals in the pedigrees of FAM > 1 (including those without a childhood cancer diagnosis) was 1.04 (female: 667, male: 697), therefore not accounting for the observed female predominance.

### Cytogenetic characteristics of ALL

Data regarding cytogenetic typing of older cases of ALL or patients with ALL diagnosed abroad could not be achieved. Cytogenetic data was available for 15/22 and 120/132 cases in FAM > 1 and FAM1, respectively. Furthermore, data on risk group was available in 16/22 and 128/132 cases in FAM > 1 and FAM1, respectively. There was no observed difference in the cytogenetic type of ALL between FAM1 and FAM > 1. However, in FAM > 1, there were no cases of high- or very high-risk leukemia. In contrast, in FAM1 21.4% of ALL (*p =* 0*.*025) were defined as high- or very high risk.

### Cancer predisposition syndromes in families with multiple childhood cancers

A cancer predisposition syndrome was present in four of the study patients in FAM > 1. Two patients with optic nerve glioma were diagnosed with neurofibromatosis type 1. One patient with ALL was diagnosed with Down’s syndrome. Interestingly, one study patient with bilateral retinoblastoma tested negative for any known hereditary mutations in the *RB* gene. However, this patient had a first degree relative with bilateral retinoblastoma and therefore both cases were defined as being hereditary.

## Discussion

Here, we show that in the LCCG population, 4.4% of childhood cancer patients have a relative with a childhood tumor amongst relatives to a 3rd degree, which is in line with previous studies [2, 3]. Interestingly, we further examined relatives to a 6th degree, which displayed a 7.8% incidence of multiple childhood cancers within a family, indicating that our study may detect heritable genetic factors of low penetrance. Both these percentages are higher than that observed in the general childhood cancer population, which supports the observations that relatives of childhood cancer patients have an increased risk of childhood cancer.

There was a difference between the percentage of leukemia cases in the full LCCG-study and in the general population. The higher proportion of patients with childhood leukemia in our cohort may be due to the inclusion of childhood cancer survivors, the majority of which are patients with leukemia. We found a lower proportion of CNS tumors in the LCCG-study compared to the general population, which could be due to lower participation rates of these patients compared to patients with other tumors (Table 1).

The majority of study patients with a childhood leukemia or CNS tumor in FAM > 1 had a relative with a matching diagnosis. This is a higher proportion than expected when compared to the general childhood cancer population. Our study is in line with previous studies that have observed a generally increased risk for cancer in relatives of children with leukemia or CNS tumors [3–5, 7, 25].

Curtin et al. 2013 [3] recently described an increased risk of childhood cancer amongst relatives of patients with childhood leukemia, however the types of diagnoses amongst relatives were not specified. The high ratio of ALL among relatives of children with leukemia in our study suggests that ALL might be responsible for a part of the increased risk showed by Curtin et al. Interestingly, no ALL case in FAM > 1 were classified as high risk, and we can speculate as to whether the different ALL risk types are related to specific genetic predispositions.

The high probability of matching diagnoses in relatives of children with childhood leukemia or CNS tumors suggests that there might be a higher degree of heredity in these diseases compared to other childhood cancer types. This is further supported by two studies on siblings with childhood cancer from 1977 and 1996, where Draper et al. observed that the number of sibling pairs where both had the same childhood cancer diagnosis was especially high for siblings with leukemia and CNS tumors [25]. However, the portion of siblings with matched diagnoses was much lower than siblings with different diagnoses. In contrast, our present study showed that a majority of the leukemia and CNS tumor cases in FAM > 1 had a relative with a matching diagnosis. This discrepancy between studies is most likely attributed to the fact that our study included distant relatives while Draper et al. focused solely on siblings. With this in consideration, our study may shed new and broader light on the potential for hereditary factors in CNS tumors and leukemia.

Three of four patients with neuroblastoma had a relative with a childhood CNS tumor. Even though the group is too small to draw any conclusions it could be of interest to study the connection between neuroblastoma and CNS tumors in a larger study. To our knowledge no such observation has previously been made.

Patients with a childhood cancer diagnosis in FAM > 1 had a significantly higher proportion of females than those in FAM1. This disparity was only observed among families with childhood leukemia and CNS tumors (Table 3). It should also be noted that the male-to-female ratio of the entire LCCG-study as well as all individuals (patients and relatives) in FAM > 1 was greater than 1, and therefore does not account for the female predominance. Previous publications have shown that infants with leukemia are predominantly female [26, 27]. However, the mean onset age of childhood leukemia in FAM > 1 in the current study was five years of age (Table 2) and only one female FAM > 1 patient with childhood leukemia was younger than 2 years (Table 1). Interestingly, in a study of cancer heredity of 1st degree siblings the Swedish population, the six twins with ALL were all female [28]. A comprehensive review of all twin cases with ALL published in 2003 gives no data on gender [29]. Another study showed that daughters to mothers with multiple sclerosis (MS) had an increased risk for leukemia [30], however no cases of MS were identified among mothers of children with leukemia in FAM > 1.

Furthermore, in a study by Magnusson et al. published in 2011, a female predominance among childhood cancer patients in FAM > 1 was also observed, however these findings were considered coincidental due to the small size of their cohort [2]. As the cohort in the current study has been substantially expanded, we can strengthen the relevance of the observed female predominance in FAM > 1, specifically amongst leukemia and CNS tumors. In addition, the fact that we observe a higher risk for female patients with either leukemia or a CNS tumor to have a relative with a childhood cancer diagnosis (18.3% and 13.6%, respectively) could potentially indicate sex- dependent risk factors. Genetic analyses on females and males with childhood leukemia or CNS tumors in FAM > 1 are now in progress.

The comprehensive Swedish national registries allow us to extend the pedigrees to 3rd degree relatives, which has previously been done in a limited number of studies [2, 3]. As this study expands up to 6th degree it increases the chance of locating hereditary factors with a low penetrance, which may explain observed trends in our study that have previously been overlooked.

The fact that only 4.6% of all eligible individuals actively declined participation is encouraging. Adding those not returning the quite extensive questionnaire, despite intent of participation and providing blood samples, we here present a participation rate of 79%. This number suggests that our cohort is representative of the southern healthcare region in Sweden. The fact that we include both newly diagnosed patients and cancer survivors in the cohort might screw the results toward survivors’ characteristics, but this should not influence the results presented here. The relative small number of families with more than one childhood cancer case is a limitation for results regarding smaller diagnostic groups.

## Conclusions

Here we show that families with a case of childhood leukemia or CNS tumor have an increased risk of having a childhood relative with the same diagnosis. That this risk is higher if the patient is female could indicate gender-specific genetic factors responsible for a heritability of the disease. This study therefore serves to identify families suitable for further genetic analyses, which are currently underway.

## Abbreviations

ALL: acute lymphatic leukemia
AML: acute myeloid leukemia
CNS: central nervous system
FAM > 1: families with multiple cases of childhood cancer
FAM1: families with one case of childhood cancer
FDR: false discovery rate
ICD-7: the International classification of diseases 7th edition
LCCG-study: Lund Childhood Cancer Genetic study
